# Glucose-Related Traits and Risk of Migraine—A Potential Mechanism and Treatment Consideration

**DOI:** 10.3390/genes13050730

**Published:** 2022-04-22

**Authors:** Md Rafiqul Islam, Dale R. Nyholt

**Affiliations:** Statistical and Genomic Epidemiology Laboratory, School of Biomedical Sciences, Faculty of Health, Centre for Genomics and Personalised Health, Queensland University of Technology, Brisbane, QLD 4000, Australia; d.nyholt@qut.edu.au

**Keywords:** migraine, glycaemic traits, glucose, insulin, type 2 diabetes, genetic

## Abstract

Migraine and glucose-related (glycaemic) traits (fasting glucose, fasting insulin, and type 2 diabetes) are common and complex comorbid disorders that cause major economic and social burdens on patients and their families. Studies on the relationship between migraine and glucose-related traits have yielded inconsistent results. The purpose of this review is to synthesise and discuss the information from the available literature on the relationship between fasting glucose, fasting insulin, and type 2 diabetes (T2D) with migraine. Publications on migraine and fasting glucose, migraine and fasting insulin, and migraine and T2D were identified from a PubMed and Google Scholar database search and reviewed for this article. Multiple publications have suggested that the comorbidity of migraine and glucose-related traits may have a similar complex pathogenic mechanism, including impaired glucose homeostasis, insulin resistance, reduced cerebrovascular reactivity, abnormal brain metabolism, shared genetic factors, neurotransmitters, and sex hormones. Furthermore, several studies have found a bi-directional link between migraine with insulin resistance and T2D. There is strong evidence for a biological association between migraine headache and glucose-related traits, and burgeoning evidence for shared genetic influences. Therefore, genetic research into these comorbid traits has the potential to identify new biomarkers and therapeutic targets and provide biological insight into their relationships. We encourage healthcare professionals to consider the co-occurrence of migraine with glucose-related traits in the evaluation and treatment of their patients.

## 1. Introduction

Migraine is a chronic neurological disease with repeated attacks of headache lasting between 4 and 72 h often accompanied by nausea, vomiting, photophobia, and phonophobia [1,2]. Migraine is considered a complex neurovascular brain disorder [2,3], and generally affects people during their most productive years (age 25–50) [4,5]. Migraine has a lifetime prevalence of 15–20%, and worldwide, it is the third most common medical disease and second most disabling neurological disease. In women, the lifetime and yearly prevalence of migraine is 33% and 18%, respectively, whereas it is 13% and 6% in men [6]. Studies conducted in the United States and Europe have shown that the rates of migraine in women (15–18%) are around three times higher in comparison to men (6–8%) [7]. Migraine is associated with a significant economic strain on patients, their families, and the community due to the loss of productivity and healthcare resource utilization [8]. Migraine, with a considerable annual expense of US $20 billion in the United States and €111 billion in the European Union, is a substantial cause for societal and economic concerns [6,9]. The International Headache Society (IHS) classifies approximately 95% of migraine headaches into two major clinical subclasses, migraine without aura (MO), affecting 70–80% of migraineurs, and migraine with aura (MA), where affected patients experience auditory, visual, and sensory hallucinations [10,11]. MA is also split into three main subtypes: migraine with brainstem aura, hemiplegic, and retinal migraine. Migraine can be categorised as episodic (EM) or chronic migraine (CM) according to the frequency of headache [12].

Furthermore, the complexity of migraine is increased by environmental factors that have been reported to play a significant role in triggering and continuing migraine in some sufferers. Environmental factors which may precipitate migraine include skipped or delayed meals, dehydration, hormonal changes, bright light, loud noise, oral contraceptives, and hormonal replacement therapies [13]. Different comorbidities like neurological, cardiovascular, psychological, and endocrine are strongly associated with migraine that can substantially affect disease progression and therapeutic and preventive approaches. In terms of cardiovascular comorbidities, the incidence of stroke [14], angina, and myocardial infarction [15], is increased in migraine (especially MA) patients, compared with non-migraine patients. The elevated risk in migraineurs for several cardiovascular diseases suggested that these pathologies are underpinned by a specific metabolic risk factor(s) and prompted numerous studies to detect common metabolic anomalies. Many studies have examined traits related to glucose metabolism (glycaemic traits). For almost a century, migraine has been associated with hypoglycaemia [16,17]. Reduced blood glucose levels have long been known to trigger or worsen migraine attacks in some patients [16,18,19,20]. Early experimental research suggests that the metabolic alterations produced by fasting or glucose or insulin administration can precipitate migraine attacks [21]. Insulin is also a vital regulator of brain glucose metabolism, and hypoglycaemia can induce migraine attacks in CM patients following prolonged fasting [22]. Other migraine studies have observed interictal impairments in glucose tolerance and insulin resistance [23,24]. Insulin is the key regulator of glucose homoeostasis, promoting glucose absorption from the blood into primarily fat and muscle cells via insulin-sensitive glucose transporters (GLUTs), in particular GLUT4 [17].

Although there are inconsistent results concerning the incidence of metabolic complications in migraine, CM patients have been reported with higher resistance to insulin [25]. Insulin resistance (IR) was found to be considerably greater compared to non-migraineurs in a study conducted in young, non-obese, and non-diabetic migraine sufferers [26]. However, IR is a key pathogenetic factor in developing type 2 diabetes (T2D). Conflicting results in various studies have been reported about the incidence of T2D in migraineurs [27,28,29]. In those patients, IR associated with CM can increase the risk of T2D that is more likely to occur when additional T2D related pathogenetic abnormalities are present [30,31,32]. Therefore, CM patients with higher IR may be more likely to develop T2D if related impairment occurs in the secretion of β-cell insulin [30,31,33]. A variety of illnesses, including dyslipidaemia, obesity, diabetes, high blood pressure, stroke, and coronary artery disease, are all associated with IR [34]. Since migraine and glucose-related traits are common disorders and often coexist, shared aetiologies can induce their comorbidity, and the combination of both conditions adds significant burdens upon society. Therefore, this narrative review will examine the relationship between migraine and glucose-related traits by focusing on observational epidemiological and genetic studies.

The most relevant observational epidemiological studies that support the shared biological mechanisms between migraine and glucose-related traits have been summarised in the first part of this study. The second section highlights shared genetics, including candidate genes and genetic variants involved in the pathophysiology of migraine and glucose-related traits. Finally, we highlight key findings and recommend future investigations on the comorbidity of migraines and glucose-related traits.

## 2. Methods

This narrative review used PubMed and Google Scholar databases to find English-language publications published up to March 2022, including original research and reviews articles. The goal of our study was to compile information from all studies (observational epidemiological studies, candidate gene association studies, and genome-wide association studies) that investigated the relationship between migraine and glucose-related traits. Search terms used “migraine” and “hypoglycaemia,” “migraine” and “blood glucose,” “migraine” and “glucose metabolism,” “migraine” and “insulin resistance,” “migraine” and “insulin metabolism,” “migraine” and “metabolic syndrome,” and “migraine” and “diabetes.” In addition, relevant articles were selected for the review according to specific headings. We also conducted an in-depth review of candidate gene association studies (CGAS) for migraine in this article (i.e., PubMed was searched using the terms “migraine” and “candidate gene study”). We then searched PubMed for overlapping CGAS for glucose-related traits (using the terms “diabetes” and “one migraine potential gene [e.g., “*MTHFR*”], and “candidate gene study”).

## 3. Results

### 3.1. Migraine and Hypoglycaemia

Hypoglycaemia (low blood sugar/glucose) is a medical condition that can trigger or exacerbate migraines and other headaches [16,35,36]. Blood glucose levels may drop too low when food intake is insufficient for the body’s needs. A similar condition can occur if someone avoids meals, maintains diet, and fasting condition. One of the first hypotheses is that hypoglycaemia possibly plays a causative role in fasting headaches. In general, serum glucose levels can be a significant and specific precipitating factor for certain migraine patients. It is widely known that lower blood glucose levels can trigger or deteriorate migraine attacks [18,20,37]. Fasting is one of the most well-known and frequently reported migraine triggers with a percentage range from 39% to 66% [13,38,39,40], and is very common in migraine patients during longer fasting [35]. In particular, some experts reported that small alterations of blood glucose might change pain receptors in the brain for some genetically predisposed individuals, which contribute to fasting headache [41]. Numerous clinical studies [13,42,43,44,45] endorse there is a well-documented link between fasting and migraine, including the studies related to fasting for religious purposes like Ramadan [46] or Yom Kippur [47]. An overnight fasting condition can trigger migraines in certain people [48]. Indeed, fasting, and missing meals can contribute to the development of migraines. For example, one study reported that migraine attacks had improved following correction of hypoglycaemia with intakes of orange juice for a 38-year-old obese woman with migraine [16]. The researchers showed the same results in four other individuals and concluded that decreased blood glucose was linked with headaches. These observations indicate that glucose deficit can trigger fasting-induced headaches directly or indirectly. Likewise, Blau and Cumings [18] noted that six of 12 patients suffered migraine headaches when fasting. Thus, the attacks might be induced by hypoglycaemia, and the ingestion of food was a simple way of preventing headaches. Furthermore, Blau and Pyke [49] once again pointed out that a significant reduction of migraine occurred due to the hypoglycaemia correction in T2D and migraine patients. Five of the 36 patients with migraine and T2D investigated by the authors resolved or reduced their migraine attacks after correction of blood glucose, and four were affected by migraine attacks by nocturnal hypoglycaemia. The migraine attack in six of the remaining 27 patients was associated with fasting or food skipping.

Clinical studies have shown that fasting result in activation of the insulin receptor. Surprisingly, intravenous insulin infusion is effective in causing migraine aura. Studies reported insulin-induced hypoglycaemia could lead to migraine-like pain since blood glucose levels dropped suddenly [19]. However, further evidence was provided a decade later with the finding that sucrose-induced reactive hypoglycaemia could trigger migraines [23]. Reactive hypoglycaemia refers to low blood sugar that is relatively uncommon, where a high-sugar meal induces hypoglycaemia, due to a rapid increase in blood sugar levels (hyperglycaemia), causing an insulin overproduction and triggering a rapid drop in blood sugar levels. Insulin is also a key regulator for brain glucose metabolism [24], and hypoglycaemia can lead to a migraine attack in migraine patients after prolonged fasting [35]. Dalkara and Kilic [41] wrote a review of the metabolism of brain glycogen during migraines. In astrocytes, the plasma glucose is stored as glycogen and quickly digested for glutamate and potassium uptake during the intensive synaptic activity of headache attacks. The authors proposed that extended periods of low blood glucose and sustained sympathetic activity in long-term fasting could diminish existing glucose derived from glycogen generated in presynaptic astrocytes and trigger aura and headache [41]. Magnetic Resonance Spectroscopy (MRS) investigations in migraines, for example, consistently demonstrate hypometabolism or reduced ATP levels due to anomalies of mitochondrial oxidative phosphorylation (OXPHOS) [50]. In addition, early investigations supporting these results indicated metabolic alterations resulting from fasting or administration of glucose or insulin can lead to migraine attacks in susceptible patients [23,24,26]. However, contrary arguments are made in ICHD-3beta [12] that state headache is not a common complaint in individuals with symptomatic hypoglycaemia. For example, insulin-induced hypoglycaemia did not produce headaches in most migraineurs studied [19]. In this study, Pearce et al. reported that headache occurred in two of the 20 individuals with migraines included in his research due to insulin-induced hypoglycaemia during a 2-h observational period. Thus, he concluded that migraine attacks might be caused by a rapid decrease in blood glucose and metabolic events [19]. Therefore, the assumption is that a sudden reduction of blood glucose levels could be attributed to migraine attacks and other metabolic events.

A hereditary state of altered glucose transport into the brain, *GLUT1* (glucose transport protein type 1) deficient syndrome, is associated with hemiplegic migraine and migraine with auras [51]. Several heterozygous mutations have been found in the *SLC2A1* encoding *GLUT1* gene. These patients have a glucose transportation deficiency across the blood-brain barrier and have different neurological abnormalities [41]. This deficient syndrome of *GLUT1* supports that glucose insufficiency can trigger migraine headaches. In addition, hyperglycaemia makes the cortex more resistant to the start of cortical spreading depression (CSD) and accelerates CSD recovery, while hypoglycaemia has the inverse effect on CSD lengths [52]. Insulin-induced hypoglycaemia dramatically prolongs the duration of CSD in experimental mice, which is thought to induce migraine aura and lead to headaches [52]. One experimental study published in 2017 found that the administration of insulin, glucagon, or leptin dramatically modulates the trigeminovascular system’s neuronal activity because of metabolic changes, which is a critical system in the pathogenesis of migraine headaches [53]. This reveals a possible neurobiological relationship between migraine and alterations in glucose homoeostasis [53]. Furthermore, recent bioinformatics analysis identified one of these single nucleotide polymorphisms (SNPs) (rs1024905, minor allele G), which is associated with increased migraine risk, is also associated with decreased expression of the *C12orf5* gene in whole blood [54], cerebellum, and temporal cortex [55]. The *C12orf5* gene was recently found to encode the TP53-inducible glycolysis and apoptosis regulator (*TIGAR*), which primarily acts as an inhibitory regulator of glucose breakdown (glycolysis) in human cells. Thus, decreased expression of *TIGAR* will result in increased glucose breakdown due to decreased blocking of glycolysis [56]. Hence, migraine sufferers carrying the rs1024905-G risk allele may also have increased glucose breakdown.

### 3.2. Migraine and Insulin Resistance

Migraine and metabolic syndrome are both prevalent and expensive conditions. Both disorders coexist, but there is an unclear relationship between the two processes. IR is one aspect of metabolic syndrome [57]. It is becoming apparent that the metabolism of insulin and glucose in migraine patients are affected and can play a pathophysiological function [57]. Furthermore, the 1-year migraine prevalence in metabolic syndrome was found to be increased to 11.9% of men and 22.5% of women who had higher body mass index (BMI), waist circumference, and a high proportion of diabetes mellitus [57]. Bic et al. [58] have proposed that biological conditions such as insulin resistance that increased free fatty acids and blood lipids could be an underlying cause of migraine headaches.

Two investigations have found that insulin sensitivity is reduced in patients with migraines, suggesting a role for insulin resistance in migraine and vascular disease comorbidity [24,26]. Clinical and epidemiological research has repeatedly linked migraine to depression, T2D, cerebrovascular diseases, and obesity, where insulin resistance is involved [59,60]. Furthermore, multiple investigations [60,61,62] have found that impaired glucose metabolism and insulin resistance are frequent pathophysiological aspects in T2D, obesity, depression, and dementia. Thus, insulin resistance may play a significant metabolic role in associating migraine and various comorbidities. When comparing the lowest to the highest quartile of homeostatic model assessment (HOMA), hyperinsulinemia is related to 5.67 times greater risk of migraine [63]. The plasma concentration of glucose in migraineurs was substantially more significant during the oral glucose tolerance test (OGTT) than in the controls. Several insulin sensitivity measures, such as ISI Stumvoll and OGIS-180, demonstrated an insulin resistance condition in migraineurs [26]. Cavestro et al. reported that both glucose and insulin were considerably higher (*p* < 0.001) in patients than in healthy controls after OGTT in a larger group of 84 migraine patients [24]. More recently, several clinical trials confirmed the incidence of insulin resistance in patients suffering from both episodic and chronic migraines [22,64,65]. Another study indicates that episodic migraine patients were normal in terms of insulin sensitivity, but chronic migraine subjects have been significantly linked to increased insulin resistance [22,66]. The risk of physical and mental illnesses such as hypertension, depression, and diabetes has increased in people with chronic migraines compared with those with episodic headaches [28]. Contrary to the results of most published studies, a few studies have not supported a relationship between migraine and IR [67,68]. However, these studies examined IR in migraineurs using fasting glucose and insulin levels rather than during a dynamic test such as OGTT. For example, one study by Sacco et al. [68] investigated 50 migraineurs with aura, 50 migraineurs without aura, and 50 controls and reported that migraine was not associated with IR.

The precise pathophysiological changes that could cause an association of migraine with IR are unclear. Brain metabolism and cerebral blood flow [69,70] are regulated by insulin through insulin receptors located in many brain parts; however, how these receptors transmit signals and exert resistance on its function remains uncertain. New research has revealed that insulin significantly influences glucose and energy homeostasis within the brain, even though the brain was long considered an insulin-independent organ [71]. According to some experts [60], peripheral insulin resistance (IR) can spread to the brain, resulting in brain insulin resistance. During times of high metabolic demand, this condition has been shown to lower insulin receptor levels in neurones and astrocytes, resulting in a decrease in glucose absorption and glycogen formation, which could trigger the neuronal cell stress associated with migraine chronification [60]. Two genetic studies have identified an important link between insulin receptor (*INSR*) gene polymorphisms and migraine [72,73]. These polymorphic gene-coded receptors, assessed in mononuclear cells, did not demonstrate in vitro functional problems but could be potentially dysfunctional in vivo, particularly in the hypothalamus and limbic areas where many receptors are expressed [74]. However, it is not clear whether the associated polymorphism in the *INSR* gene causes loss or increased function. Furthermore, insulin may be implicated in migraine pathophysiology because it is linked with glucose metabolism and directly impacts gonadotropin secretion through the hypothalamus [69]. Studies in laboratory rats have demonstrated that insulin induces the secretions of gonadotropins from the hypothalamic and pituitary glands [75]. The hypothalamus’ central involvement in autonomic function and homoeostasis indicates that it may contribute to some autonomic symptoms linked with migraine [76,77,78] or its prodromal phase [77,78]. Activating the brainstem regions and hypothalamus before the onset of pain shows that these structures play an important role in migraine’s pathophysiology [79,80]. Positron emission tomography (PET) showed for the first time that spontaneous migraine attacks activate the hypothalamus and demonstrate that the hypothalamus is implicated relatively early in a migraine attack [80]. Estroprogestin medications also cause hyperinsulinism and hypoglycaemia, aggravating migraines in individuals who receive these medicines [81]. This connection may explain the strong links between insulin and female sex hormones and the deterioration of migraines due to estroprogestins, and it could support the theory of menstrual migraines [24].

Calcitonin gene-related peptide (CGRP) is well known to be involved in migraine pathophysiology [82,83], with serum concentrations of CGRP being shown to be increased during migraine attacks [84], resulting in sensitivity of the trigeminal system [85]. Besides its role in migraines, animal studies revealed an association between CGRP and the modulation of insulin and glucagon production [86,87]. Therefore, higher plasma insulin levels in migraine patients can also be caused by CGRP, a neuropeptide expressed in sensory nerves associated with glucose metabolism and plays a vital role in migraine pathogenesis [88]. Furthermore, CGRP antagonism via receptor antagonists or by monoclonal antibodies reduces and prevents migraines [89]. CGRP is known to induce resistance to insulin and obesity [90] by decreasing the release of insulin from β-cells [91]. One study reported that CGRP is higher in obese and diabetic Zucker rats, and administration of capsaicin, a sensory nerve blockade, reduces blood glucose following an oral glucose tolerance test and enhances the sensitivity to insulin [92]. These authors later revealed that the sensory neurons of the pancreatic islets carrying CGRP are sensitive to capsaicin. Capsaicin inhibits them with a consequent positive effect on the secretion of insulin and glucose tolerance [86].

### 3.3. Migraine and Diabetes Mellitus

Diabetes mellitus (DM), usually referred to as diabetes, is a chronic lifelong disorder caused by reduced pancreatic production of insulin or the inefficiency of the generated insulin, referred to as insulin resistance. This deficiency leads to increased glucose concentrations in the blood, damaging many systems of the body. There are two primary forms of diabetes: type 1 diabetes (T1D), an autoimmune disease that occurs most often in children and adolescents, and T2D due to the body’s failure to respond adequately to insulin triggered by the pancreas. T2D impacts all ages, with a global prevalence of 9.3% [93]. The possible association between migraine and diabetes remains unknown due to conflicting findings. Some studies reported diabetes to have a protective effect for migraine, some studies suggest diabetes increased the risk for migraines, while other studies report no association between the two conditions.

Migraine is also linked to insulin resistance, suggesting a relationship between migraine and T2D. Several studies have shown that disabled insulin sensitivity in migraines contributes to the possibility of increasing the risk of T2D for migraine patients [29,94,95]. Population-based cross-sectional analyses were conducted by Bigal et al. [27], which was included in the American Migraine Prevalence and Prevention (AMPP) study demonstrated that people who had a migraine in the previous year were more likely than headache-free controls to have T2D (12.6% versus 9.4%; odds ratio (OR) = 1.4, confidence interval [95% CI: 1.2–1.6]). However, the prevalence of diabetes among patients with episodic and chronic migraines did not differ in the same populations [28].

In contrast, some studies have documented an opposite association between diabetes and migraine [96,97,98,99,100]. For example, Nord–Trøndelag Health Surveys [99] indicated that type 1 diabetes (T1D) is associated with decreased migraine prevalence (OR = 0.47, confidence interval [95% CI: 0.26–0.96]), and any other type of headaches (OR = 0.55, [95% CI: 0.34–0.88]) compared to a patient without DM. While many studies did not distinguish between T1D and T2D, a cross-sectional analysis of the HUNT study data [96] indicated that the prevalence of migraine in both types 1 and 2 DM is less likely to be reported than individuals without DM in the last year (multivariable-adjusted OR = 0.4, confidence interval [95% CI: 0.2–0.9] for T1D and migraine and multivariable-adjusted OR = 0.7, [95% CI: 0.5–0.9] for T2D and migraine). In addition, the Norwegian cohort study [100], consisting of men and women under 80 years of age, demonstrated that both types 1 and 2 DM were related significantly with reduced risk of migraine (OR = 0.74, confidence interval [95% CI: 0.62–0.89]) and (OR = 0.86, confidence interval [95% CI: 0.80–0.92]). This result is in line with other findings from an old clinical survey [49] by Blau and Pyke that shows that the onset of diabetes greatly reduces the incidence of migraine attacks in migraine patients.

**Figure 1:**
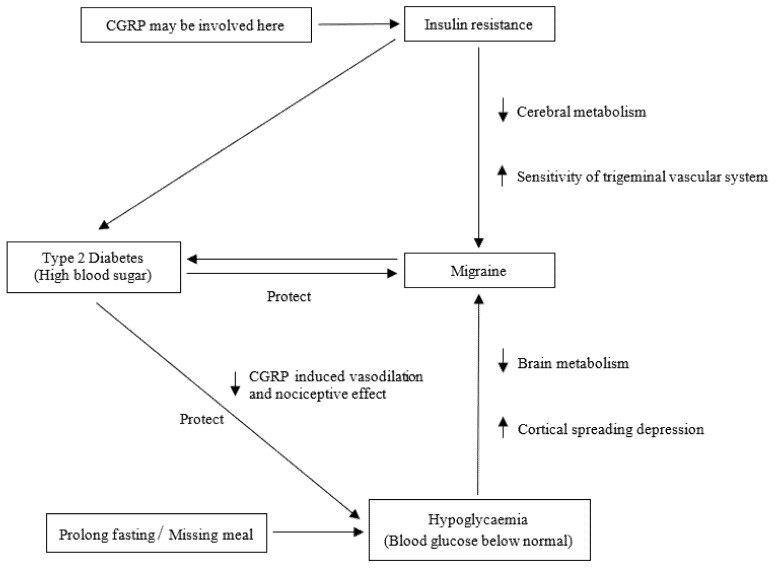
**Possible mechanisms describing the relationship between migraine and glucose-related traits.** Cortical spreading depression (CSD) in migraine stimulates pain pathways originating in the parasympathetic trigeminal nerve fibres, resulting in the production of calcitonin gene-related peptide (CGRP) [52,84,85]. It is suggested that CGRP expressed in sensory nerves associated with glucose metabolism and plays a vital role in migraine pathogenesis [88]. A change in blood glucose level is another significant aspect in this association where hyperglycaemia makes the cortex more resistant to the start of CSD and accelerates CSD recovery. At the same time, hypoglycaemia has an inverse effect on CSD length [52]. Low blood glucose and sustained sympathetic activity in long-term fasting could diminish existing glucose derived from glycogen generated in presynaptic astrocytes and trigger aura and headache [41]. In migraine, magnetic resonance spectroscopy (MRS) investigations demonstrate hypometabolism or reduced ATP levels due to mitochondrial oxidative phosphorylation (OXPHOS) anomalies, which can lead to migraine attacks [50]. In diabetes, sensory nerve damage due to lower CGRP expression may have diminished CGRP-induced vasodilation and nociceptive effects, which can explain the decreased prevalence of active migraine [101].

Likewise, analysis of data from the Women’s Health Study (WHS) cohort [29] of women without DM at baseline revealed that individuals with a history of migraine, active MA, or active MO were at reduced risk (OR = 0.79, confidence interval [95% CI: 0.67 to 0.94]) of acquiring T2D as compared to non-migraineurs women. In addition, Fagherazzi et al. [101] examined the association between migraine and drug-treated T2D in French prospective cohort research. This study has shown a lowered risk for developing T2D for women with active migraines and a decrease in the prevalence of migraines in patients with T2D. Notably, the prevalence of migraine in this cohort reduced from 22 to 11 percent during the 24 years prior to T2D diagnosis, which implies that diabetes may protect against migraine. Early case-control research [98] similarly revealed a reduced migraine frequency in people with DM compared to individuals without DM. A cross-sectional study [97] in Norway based on an analysis of data on the prescription of medicines produced similar conclusions. Compared to the non-diabetic group, the study found that people with DM had an overall reduced prevalence of medically-treated migraines and the pattern became more evident with increasing age. Age may also be a significant effect modifier in the relationship between T2D and migraine, indicating an opposite association among older people [100]. However, large-scale population-based investigations have revealed no statistically significant difference in migraine incidence between children with T1D versus controls [102]. Similarly, studies by López-de-Andrés et al. [103] and Haghighi [94] showed that migraine prevalence between those with and without diabetes is not significantly differentiated. It is particularly interesting to see the relationship between headache and DM, as the DM has an impact on vascular reactivity [104,105], and causes diabetic neuropathy [106], and contributes to changes in the style of life that can all be significant in migraine pathophysiology. Migraine and diabetes both have lifestyle-related factors that play a role in their aetiology and pathophysiology [107]. Lifestyle changes can alter the functioning of both central and autonomic nerve systems, which can lead to migraine attacks or diabetes. A higher incidence of migraine attacks is associated with smoking, alcohol consumption, and sedentary life in people with diabetes [107]. Several studies have found that migraine sufferers may benefit from weight loss and lifestyle slimming techniques, including physical activity, exercise, and sports [107,108]. At present, the specific biological mechanisms behind this apparent protective effect of diabetes on the risk of developing migraine attacks are unknown. Considering the preceding discussion, the probable biological mechanisms of comorbidity between migraine and glucose-related traits can be found in Figure 1.

### 3.4. Migraine and Glucose-Related Traits: Lifestyle Changes and Treatment

A low-glycaemic diet may be a beneficial and effective approach in treating migraine headaches [109]. This diet has been suggested as a way to reduce inflammation [110]. A diet high in fat and low in carbohydrate (ketogenic diet) works similarly to fasting, where ketone bodies are raised and can be utilised as an alternate energy source to repair anomalies in glucose metabolism observed in migraines [111]. The idea that migraines result from insufficient brain energy or uncompensated oxidative stress has reintroduced the ketogenic diet into the spotlight. A low-calorie ketogenic nutritional plan is widely used for weight loss, as well as for the treatment of various pathologies, including neurological conditions [112]. This diet has recently demonstrated encouraging results in migraine prevention, potentially influencing numerous pathophysiological systems [113]. For instance, the ketogenic diet can improve mitochondrial function, reduce oxidative stress, lower cerebral excitability, suppress inflammation and decrease cortical spreading depression [111]. A recent double-blind, crossover study by Di Lorenzo et al. compared the preventative effects of a very-low-calorie ketogenic and non-ketogenic diet for one month in 35 overweight migraine sufferers. The ketogenic diet was significantly more effective than the non-ketogenic diet in lowering monthly migraine days. For example, the 50% responder rate for monthly migraine days was 74.28% (26/35 patients) during the ketogenic diet, but only 8.57% (3/35 patients) during the non-ketogenic diet [114]. In addition, a longitudinal study found that following nutritional advice based on the Healthy Eating Plate (HEP), especially reducing carbohydrate, red and processed meat consumption, was associated with a reduction in migraine frequency and disability [115]. Given lifestyle modifications can be effective in migraine therapy, especially when migraine is associated with other diseases such as glucose-related traits, primary care physicians have developed SEEDS (Sleep, Exercise, Eat, Diary, and Stress) guidelines to assist migraine patients change their lifestyle components [111].

### 3.5. Shared Genetic Basis

Twin and family studies provide numerous approaches to determine familial aggregation, environmental contributions, and genetic influences on disease. Classical twin research compares the similarities between monozygotic (MZ) twins to dizygotic (DZ) twins, whereas familial studies normally include parent-offspring pairs or sibling pairs. Genetically linked individuals (for example, twins) can increase the power of genetic research to detect the genetic variations potentially related to complex diseases [116]. Family and twin studies have shown that migraine tends to occur in families, and there are robust genetic components that can lead to the development of migraine in an individual, familial hemiplegic migraine (FHM) is the best example because it is a severe and rare monogenic form of MA which follows an autosomal dominant pattern of inheritance [117]. Higher concordance of disease rate in monozygotic twins than dizygotic twins have been consistently found in twin studies, indicating a significant genetic contribution to migraine [118,119,120,121,122,123]. The estimated heritability of migraine is 33–65% (depending on migraine-type—MA or MO) [118,124,125]. The heritability estimate describes how much of the phenotypic variation of migraine in a population is due to genetic factors. Even when twins have grown apart, the increased concordance of migraine among MZ twins continued [126,127,128,129,130], undermining the possible effects of common (shared) environmental factors and thus supporting the prominent roles of genetics and non-shared environmental factors in the aetiology of the disease.

Intense genetic research has also been performed on glucose-related traits such as fasting glucose, insulin measures, and glycated haemoglobin (HbA1c), used to diagnose, and control T2D, which are also important risk factors for migraine even in the non-diabetic range. Genetic and environmental factors influence the fasting blood glucose (FPG) level. Higher than optimal levels of FBG can also result in increased morbidity and death, even if less than the diagnostic diabetes threshold (FBG ≥ 7.0 mmol/L is the diagnostic criterion for diabetes) [116]. The chance of developing microvascular illness, such as heart attack or stroke, starts to rise long before this diagnostic threshold [129]. Diabetes and higher than optimal FPG levels combined resulted in 3.7 million deaths worldwide between 1980 and 2014 [116]. The heritability for fasting glucose ranges from 10–75%, and for fasting insulin, it is 20–55% [130]. Twin and family research have long indicated the genetic components to the susceptibility of T2D. T2D runs in families, and genetic as well as environmental factors affect the likelihood of developing T2D disease [131]. Heritability estimates of T2D range from 25% to 80% [131]. These differences in heritability may be linked to the study designs and/or reflect significant variations as heritability can depend on gender, age, or background [130]. Furthermore, twin studies show very strong concordance (70%) for MZ twin pairs, whereas the concordance in DZ twins is only 20–30% [131].

Migraine and glucose-related traits can have a shared genetic basis. Several migraine comorbidities are explained by common genetic risk factors [132,133,134]. This concept can explain some of the associations between migraine and glucose-related traits. A range of metabolic and nociceptive processes are modulated by certain neurochemicals, including nitric oxide and CGRP. Genetic risk factors that contribute to insulin resistance and impaired glucose metabolism may also lead to migraine headaches.

Although twin and family studies indicate substantial evidence for genetic components in migraine and glucose-related traits, no twin studies have examined their shared heritability (genetic correlation). However, in 2020 Siewert and colleagues [135] performed cross-trait linkage disequilibrium score regression (LDSC)—an approach to estimate trait heritability and genetic correlation from genome-wide association study (GWAS) results [136]—between migraine and multiple traits and reported a significant study-wide genetic correlation (*p* < 1.06 × 10^−3^) between migraine and T2D. Although subsequent Mendelian randomisation analysis did not indicate a causal effect of T2D on migraine, the observed genetic correlation suggests shared genetically determined mechanisms contribute to their co-occurrence—e.g., neurovascular mechanisms associated with T2D may contribute to migraine risk. Thus, identifying specific shared genetic risk factors for migraine and glucose-related traits has potential to advance our understanding of the comorbid pathophysiology between them and can be accomplished using two fundamental approaches: candidate gene association studies and genome-wide association studies.

### 3.6. Candidate Gene Association Studies

In genetic association research, candidate gene studies have identified risk variants related to a specific disease. CGAS are comparatively cheap and easy to perform, focusing on selecting genes that have previously been linked to the disease somehow. The candidate gene approach starts with selecting a putative candidate gene based on its relevance to the known or hypothesised disease mechanism under investigation. For example, the study of candidate gene associations found that about 200 genetic variations in around 100 genes can be related to a more common form of migraine [137]. These findings, however, have been inconsistent and proven difficult to replicate. Researchers have worked over decades to uncover the role of genetics in glucose-related traits using epidemiology research, candidate gene investigations, and family linkage studies. CGAS also identified genes associated with glucose-related traits before genome-wide association studies (GWAS), but many of these findings were not replicated [138,139,140,141]. Nonetheless, several candidate genes have been thoroughly examined, including those involved in the pathways hypothesised to be pathophysiology related to both disorders. Interesting overlapping candidate genes for migraine and glucose-related traits are *MTHFR*, *INSR*, *TNF*, *ESR1*, *NOS3*, and *PON1*. The overlapping candidate genes suggest potential common biological mechanisms behind migraine and glucose-related traits. Table 1 outlines some of the candidate genes that are associated with migraine and glucose-related traits.

#### 3.6.1. Methylenetetrahydrofolate Reductase

Methylenetetrahydrofolate reductase (MTHFR) transforms 5, 10-methylene tetrahydrofolate to 5-methyl tetrahydrofolate, an essential part of folate and homocysteine metabolism. The most important genetic variant is the *MTHFR* C677T polymorphism that causes hyperhomocysteinemia. The C677T polymorphism is a C to T transition at base pair 677, linked to reduced MTHFR activity as this transformation leads to an amino acid change from alanine to valine. The *MTHFR* C677T polymorphism can reduce enzyme activity by 65% and raise plasma total homocysteine levels, especially in the presence of low dietary folate. Homocysteine levels were found to be higher in migraine patients with aura [189]. Hyperhomocysteinemia (high level of plasma homocysteine) can lead to endothelial dysfunction, which in turn could contribute to the development of cortical spreading depression (a process involved in migraine aura pathogenesis) [189]. High homocysteine levels are linked with T2D via insulin resistance as homocysteine was found to have adverse effects on β-cell glucose metabolism and cell viability, thereby impairing insulin secretory activity [147]. Previous epidemiological research also indicates that migraine [190,191] and T2D [147,148] can be related to the C677T genetic polymorphism of *MTHFR*.

Rubino et al. [142] performed a meta-analysis comprising 2961 migraineurs (2170 patients with MA and 791 patients with MO), linking the *MTHFR* C677T polymorphism to migraine, and found evidence that *MTHFR* was associated with MA only (OR = 1.30, 95% CI [1.06–1.58]). Likewise, the meta-analysis carried out by Schurks et al. [143] found a similar observation that an elevated risk of MA (OR = 1.48, 95% CI [1.02–2.13]) was linked to the *MTHFR* 677TT genotype among non-Caucasians. Furthermore, Samaan et al. carried out a meta-analysis [144], which included five Caucasian datasets, showing that the TT genotype was associated with both MA and MO in non-Caucasians, whereas for the Caucasians, this variant was associated only with MA. In contrast, several investigations have reported the TT genotype to be associated with a reduced risk for MA [192,193]. However, a German study [145] with 656 MA patients and 625 controls did not supported the association between *MTHFR* TT genotype and MA, and similar results were observed in a Finnish study [146] comprising 898 MA and 900 controls.

According to recent research, there is a link between C677T and T2D susceptibility. A meta-analysis by Zhu et al. [147] was undertaken in 2014 in China better to identify the function of C677T polymorphism in T2D. This meta-analysis comprised 4656 T2D cases and 2127 controls from 29 studies, and they identified a significant relationship between the polymorphism of *MTHFR* C677T and T2D. Wang et al. [148] also observed in a study carried out in China in 2014 that C677T polymorphism in *MTHFR* can contribute to the risk of T2D. A further study by Khalid et al. [149] noticed that the relationship of *MTHFR* C677T and T2D was significant with the Arab population. However, Errera et al. [150] found no evidence for an association between the 677TT polymorphism of *MTHFR* and T2D in Brazilian populations in 2006. Similarly, no association was found for *MTHFR* C677T polymorphism in T2D development in case-control research in the Brazilian population with 47 T2D cases and 78 controls [151].

#### 3.6.2. Insulin Receptor

The insulin receptor (*INSR*) is a membrane protein on every cell’s surface and part of the tyrosine kinase receptor family [194]. The insulin receptor gene consists of 22 exons and 21 introns and spans about 120 kilobases, which maps to the short arm of 19p13.3–p13.2 chromosomes. The mature *INSR* is a hetero-tetramer that consists of two α-subunits and two β-subunits. Exons 1–11 code for the receptor’s α-subunits, while exons 12–22 code for the receptor’s β-subunits. Exon 2 codes the insulin-binding domain of the insulin receptor protein, while the protein tyrosine kinase domain needed for the insulin action is encoded by exons 17–21 [194]. *INSR* regulates insulin’s activity on target cells. Insulin’s metabolic function is governed by a series of chemical events that begin with insulin attaching to its receptor [195]. When the insulin signalling pathway is activated, the glucose transporter 4 (*GLUT4*) is translocated to the cell surface, allowing glucose to enter the cell. Thus, genes in the insulin signalling pathway, such as the insulin receptor (*INSR*), insulin receptor substrates 1 and 2 (*IRS1* and *IRS2*), and *GLUT4*, are attractive candidates for insulin resistance [196]. However, the precise mechanisms for mediating insulin resistance remain unclear. Any defect in *INSR* might lower the insulin’s action and lead to resistance to insulin, resulting in T2D [197].

In many different populations, the function of the *INSR* Val985Met variant in the predisposition to disease has been investigated. However, the findings remain inconclusive, with some research showing relevance for this variant [154,155], while others do not support this finding [156,198]. One study conducted in a Danish population found no significant association between Met985 polymorphism and T2D [156]. In contrast, the Met985 polymorphism was found at a higher frequency in T2D people (5.6%) compared to control (1.3%) participants (OR = 4.49, 95% CI [1.65–12.26]; *p* = 0.005) in a Dutch population sample recruited in Rotterdam [155]. Another study in the Netherlands, comprised of a sample gathered from Hoorn, found that the occurrence of the Met985 polymorphism in T2D patients (3.7%) and control subjects (2.7%) were similar [154]. However, a joint analysis of data from Hoorn and Rotterdam indicated a significant association between the Met985 polymorphism and T2D with a frequency of 4.4% in T2D cases compared to 1.8% in controls [154].

Factors contributing to insulin resistance and T2D have also been linked to migraine. McCarthy et al. identified polymorphism of five single-nucleotides in the insulin receptor gene in 16 North American families that were significantly associated with typical migraines, implying that the insulin receptor may play a role in the disease’s pathogenesis [72]. In addition, they have reproduced a few of their findings in an independent Australian study of 255 patients with MO and MA and 237 controls. In this investigation, 16 polymorphisms in the insulin receptor gene were genotyped in two Caucasian samples comprising a total 827 North American MA and MO patients and 765 controls. MA was most strongly linked to one exonic and two intronic variants (rs1799817, rs2860172, and rs2860174, with allelic *p*-values of 0.008, 0.002, and 0.007, respectively) in one of the samples of North American origin. In the combined study of both American samples, rs2860174 that was the only variant which was significant regardless of sex. Similar results from a large sample in a German population were reported by Netzer and colleagues [73], in which one of the five SNPs (rs2860174) showed allelic association at a significant level (*p* = 0.005). However, an Australian study [154] on one large family and a Finnish linkage study [153] on 72 families found no linkage between the region of *INSR* and migraine with aura.

#### 3.6.3. Tumour Necrosis Factor

Tumour necrosis factor (TNF) is a major pro-inflammatory cytokine that initiates and regulates the chain of events that leads to an inflammatory response [199]. TNF (previously called TNF-α) and lymphotoxin-α or LT-α (previously called TNF-β) are members of the same TNF superfamily and are coded by the same gene cluster [200]. The promoter variants −308 A/G and −238 G/A in the *TNF-α* gene (*TNF*) influence transcriptional regulation of the gene coding for LT-α (*LTA*) [200]. *TNF* has a polymorphism at position −308 in the promoter region, with either a G (TNFα1) allele or an A (TNFα2) allele [199]. The TNFα2 allele has been associated with several autoimmune diseases as it is a stronger transcriptional activator relative to the TNFα1 allele, resulting in higher levels of *TNF* [199].

An Italian study, for example, Rainero et al. [199], discovered an association between migraine and the −308 (G/A) polymorphism in *TNF*, located in the human leucocyte antigen (HLA) class III region. In MO patients (OR = 3.30, *p* < 0.001) the *TNF* −308 (G/A) polymorphism has been linked to the migraine occurrence, with individuals homozygous for the G allele have an increased risk for migraine. In a similar study from Iran, Mazaheri et al. [157] found that MO patients had a higher frequency of the −308 A allele than the control subjects (40.6% versus 22.3%, OR: 3.73, confidence interval: [95% CI 2.4–5.82], *p* < 0.0001), and thus suggest that *TNF* or a locus in linkage disequilibrium (LD) with *TNF* could be involved in the risk of migraine.

Increased TNF-α concentrations are closely associated with hyperinsulinemia and body mass index (BMI) [163]. TNF-α can disrupt insulin release and the insulin effect by interfering with insulin signalling pathways and affecting the synthesis, secretion, and activities of other cytokines. TNF-α expression in adipose tissue has been linked to insulin resistance, which is considered a key pathogenic mechanism in the development of T2D [163]. Several research investigating the relationship between *TNF* polymorphism with insulin resistance and T2D risk have been published in various countries [158,159,161]. The conclusions of these investigations, however, are mostly inconsistent. For example, Day et al. [162], found a significant association between the *TNF*-238G/A polymorphism and a decrease in insulin resistance in UK populations. At the same time, Valenti et al. [201] reported that patients with the *TNF* −238 A allele had higher levels of insulin resistance and higher incidence of impaired glucose tolerance. In response to a fat-rich meal, the −308 G/A *TNF* polymorphism is independently associated with fasting glucose concentration, postprandial triacylglycerol levels, and BMI [202]. In 2014, a meta-analysis carried out by Zhao et al. [158] showed that the polymorphism *TNF* −308 A allele in T2D increased by around 21%. Similarly, Golshani and his colleagues [174] discovered, in Iranian populations, a greater risk of T2D development associated with *TNF* −308 G/A genotype. A further study found that the *TNF* −308 A/G polymorphism was significantly associated with a higher risk of T2D in the Chinese Han population [160]. In contrast, Feng et al. [161] conducted a meta-analysis and found no significant association between the *TNF* −308 G/A polymorphism with T2D risk in Caucasian and Asian populations. In the same context, the findings of two small-sample meta-analyses have revealed that *TNF* −238 G/A was not associated with T2D [163,164].

#### 3.6.4. Oestrogen Receptor 1

In humans, there are two oestrogen receptor (ESR) isoforms: oestrogen receptor α (ESR1) and oestrogen receptor β (ESR2) [165]. ESR1 appears to be the dominant oestrogen receptor. Apart from the reproductive system, Oestrogen receptor 1 (*ESR1*) is a gene found on chromosome 6q25.1 that is expressed in numerous tissues and organs, including bone, intestinal tract, macrophages, and vascular endothelial cells. The most widely studied polymorphisms of the ESR1 gene are Pvull (−397 T>C, rs2234693) and Xbal (−351 A>G, rs9340799) [165]. Recent research has suggested that the genetic variants of *ESR1* gene have a role in the development of migraine [168]. In two separate Australian case-control populations [168], the G594A SNP in exon 8 of *ESR1* was positively associated with to migraine. In the same populations, an investigation of the progesterone receptor (*PGR*) also revealed an association. Moreover, a combined study of both hormonal genes revealed that the insertion of the Alu allele in intron 7 (PROGINS) combined with the 594A *ESR1* allele increased the risk of migraine by 3.2 fold [169]. However, follow-up investigations of two other *ESR1* polymorphisms in intron 1 and exon 4 (G325C) by the same group, found no association [203]. This is contrasted with research in a Spanish population [171] that found an association with the G325C polymorphism but not with the original G594A polymorphism. A bigger Finnish cohort study [146], examining 26 SNPs across the ESR1 gene, found no correlation with migraine, as did another Spanish study that looked at three SNPs and found no association.

Findings from several studies indicate migraine is more common in women during adolescence [204] and can be altered in different reproductive stages such as pregnancy, menopause, menstrual cycle, and hormone therapy [205,206]. This strongly suggests that fluctuating hormone levels, especially oestrogen, may play a role in precipitating a migraine attack. Previously, it has been assumed that prolonged exposure to high oestrogen levels before reducing in concentrations (oestrogen withdrawal) can cause migraine occurrence [207]. Furthermore, vascular effects of steroid hormones, like the modulation of nitric oxide generation and, hence changing vascular tone [208]. This all can be related to the pathophysiology of migraines. Schurks and colleagues [170] reported association between migraine and G594A and C325G polymorphisms of *ESR1*, which were shown to be dominant and recessive, respectively. Oterino et al. [209] conducted a study involving a multi-locus investigation of five polymorphisms related to oestrogen to resolve some of the contradicting results on *ESR1*, finding *ESR1* and *ESR2* polymorphisms association with MA/MO, but *FSHR* polymorphisms association with only MA at a nominal significance level (*p* < 0.05). These findings support the idea that hormones and/or hormone-related genes may play a role in migraine risk.

Recent research has reported that variants in *ESR1* have a significant role in developing T2D and metabolic syndromes; however, the results were conflicting [210]. The *ESR1* gene has a critical role in metabolic homeostasis. In animal studies, male and female *ESR1* gene knock-out mice have acquired metabolic syndrome characteristics including obesity due to decreased fatty acid oxidation, impaired glucose tolerance, and reduced insulin sensitivity [210]. Although a study of 47 Caucasians [166] with T2D reported no substantial difference in the allelic frequency of Pvull polymorphism of *ESR1* between type 2 diabetic and control groups, there was, however, a significant association between Pvull polymorphisms and T2D in African-Americans, European-Americans [166,167], Hungarians [211] and Egyptian women [212]. In 2018, a meta-analysis of eight studies found that T2D was associated with Pvull (OR = 0.673, 95% CI: [0.550–0.823]) but not the Xbal polymorphism. In this study, the Pvull C allele was associated with a decreased risk of T2D in Chinese, whereas the Xbal G allele was associated with a decreased risk of T2D in Caucasians [165]. Wei et al. [138] reported that the rs722208 variant of *ESR1* was associated with fasting plasma glucose (FPG) (*p* = 0.045) in Han Chinese T2D patients by analysing the association between candidate gene mutations and quantitative traits related to metabolic syndrome. Another study [213] reported that the rs2207396 *ESR1* polymorphism was associated with an increased risk for T2D in hypogonadal men.

#### 3.6.5. Nitric Oxide Synthase 3

Genes encoding for endothelial function are interesting candidate genes for T2D and migraine. Nitric oxide (NO) is a crucial endogenous endothelial-derived relaxing factor produced from L-arginine by a group of enzymes known as neuronal synthase (NOS1), inducible synthase (NOS2), and endothelial synthase (NOS3) [180]. NOS1 and NOS3 are most likely to contribute to whole-body NO generation [180]. The *NOS3* gene, located on 7q35–36, is primarily expressed in endothelial cells [180]. Many studies recently linked *NOS3* gene polymorphisms with an increased risk of migraine, insulin resistance, and T2D; however, the findings were not always conclusive.

DM and its consequences were also linked to impaired NO production [214]. Among the several functions of NO, Pieper [215] concluded that NO alterations play a major part in developing insulin resistance and T2D by showing its capability to modulate peripheral and hepatic glucose metabolism and insulin production. Several investigations have been performed to identify the relationship between T2D and *NOS3* polymorphism [175,176,180]. The research found that the 4a allele for the 4b/4a VNTR increases the risk of developing type 1 and type 2 DM [175,176,177]. Furthermore, this allele was related to endothelial dysfunction in diabetic individuals, implying that the 4a allele reduces NO bioavailability and contributes to diabetes susceptibility [214]. In addition to the 4a allele, the Asp allele for polymorphism Glu298Asp related to diabetes risk [176,180], and it appears that this association is especially important for obese individuals [181]. The *NOS3* −786 T>C and Glu298Asp polymorphisms were also linked with diabetic nephropathy [216].

Genetic susceptibility to migraine has been linked to the polymorphism in the *NOS3* gene. Despite diverse pathogenetic processes in migraine, there is compelling evidence that NO plays a critical role in migraine pathogenesis [217]. NO is a key mediator in modulating cerebral blood flow and leads to the activation of nociceptors in the trigeminovascular system during a migraine episode [217]. In this context, migraine risk was associated with the variant genotypes for −786 T>C polymorphisms [178]. In comparison to the controls, the Asp/Asp genotype for the Glu298Asp polymorphism was related to a 2-fold increase in migraine risk and a 3-fold increase in migraine with aura risk compared to migraine without aura patients [179]. Furthermore, the Glu298Asp polymorphism was associated with headache pain intensity and the age at which migraines begin [178]. Other research, however, found no association between *NOS3* polymorphisms and migraine [147,172]. While one study found no link between *NOS3* haplotypes and migraine [174], another more extensive analysis included variants for the −786 T>C, −665 C>T, 4b/4a VNTR, and Glu298Asp polymorphisms, as well as the tagSNP rs743506, found interesting results [173]. This study observed that *NOS3* rs743506 and *NOS2* 2087G/A interact significantly in patients with migraine compared to control (*p* < 0.05), and this combination impacts the susceptibility to migraine [173]. The haplotypes C-C-4a-Glu-G and C-C-4b-Glu-G were identified more commonly in women with MA than for women with MO [172]. Thus, this study suggests that *NOS3* haplotypes may influence the sensitivity to aura in migraine patients despite the lack of associations between *NOS3* haplotypes and migraines [172].

#### 3.6.6. Paraoxonase 1

Paraoxonase 1 (PON1), a polymorphic enzyme coded for the *PON1* gene on chromosome 7q21.3, has a vital function in the metabolism of many organophosphorus products, such as pesticides, neurotoxins, and aryl esters, and is mainly generated in the liver [182]. Two nonsynonymous polymorphisms, 8638 bp apart, were shown to influence *PON1* activities: substitutions of leucine to methionine at position 55 (55L/M, rs854560, c.220 T>A as per GenBank accession number NM 000446) and substitution of glutamine to arginine at position 192 (Q192R, rs662, c.632 A>G at GenBank accession number NM 000446). At position 192, the R allele was associated with rapid hydrolysis of paraoxon, while the Q allele was associated with slow hydrolysis [182]. The *PON1* gene contains many polymorphisms both in the gene’s coding regions and promoter regions, some of which (e.g., *PON1* 192Q/R and 55L/M) can alter the activity of *PON1*’s enzymes and peptide concentration [183]. In addition, PON protein possesses *PON1* and arylesterase (ARE) activity and is involved in preventing LDL oxidation and endothelial dysfunction, both of which have been linked to the pathogenesis of migraine [183]. Garcia-Martin et al. investigated *PON1* polymorphisms (*PON1* 192Q/R and 55L/M) and their association with migraine risk in 197 Spanish Caucasian migraine patients [182]. They observed that the risk of migraine in those patients is not associated with *PON1* polymorphisms, however, the genotype *PON1* 192Q/Q and allelic variant *PON1* 192Q were significantly more common in patients who had a migraine earlier in life [182]. In a subsequent investigation, biochemical assays were used to analyse *PON1* enzyme activity levels in a Turkish population of 104 migraine patients and 86 healthy participants to determine the risk of acquiring migraine [183]. The *PON1* serum activity has shown a significant drop in migraine patients. In this example, the authors genotyped *PON1* 55L/M and 192Q/R polymorphisms but found no significant differences in allele frequencies between patients and controls [183]. Similarly, an Italian study [184] demonstrated no association for *PON1* 55L/M and 192Q/R polymorphisms in 96 patients with chronic migraines.

It has been suggested that oxidative stress is responsible for impaired insulin action [218]. In the *PON1* gene locus, Met-Leu 54 polymorphism is associated with insulin resistance in healthy persons and is strongly LD with the *PON1* 192 polymorphism [219]. Barbieri et al. [219] concluded that the genotype L/L PON’s presence is associated with a higher degree of insulin resistance (IR). Insulin resistance is well documented to play a key role in the pathogenesis of T2D [197]. In various investigations, decreased *PON1* levels were identified in DM [220,221]. In one study, together with 110 healthy volunteers, a total of 86 T1D and 246 T2D patients were investigated by Flekac et al. [185]. They found that having the *PON1*-55 MM and *PON1*-192 Q/Q genotypes in diabetic patients instead of L/L and R/R genotypes, respectively, were related to poorer diabetes control. In contrast, better control of diabetes was identified in patients with L/L and R/R genotypes [185]. Several other investigations have found similar results [186,187]. For diabetic patients, Flekac et al. reported that paraoxonase levels are low in individuals with comorbidities like nephropathy, neuropathy, and retinopathy [185]. A further study conducted by Van den Berg et al. analysed 566 members of the Cohort study of Diabetes and Atherosclerosis Maastricht (CoDAM) and discovered that the R/R-phenotype was significantly more common in newly diagnosed T2D patients than in subjects with normal glucose tolerance [188].

### 3.7. Genome-Wide Association Studies

GWAS is an agnostic strategy to examine genetic variants (most often SNPs) spread across the genome for association to a particular disease or trait [222]. GWAS have revealed distinct genomic risk loci containing genetic variants associated with migraine [223,224,225] and glucose-related traits [226,227]; and the list of loci associated with each specific disorder grows with each subsequent study, mainly due to the increase in power of GWAS when performed in larger GWAS samples. So far, at least 170 genetic variants (123 genomic loci) have been associated with migraine (36), 403 genetic variants (243 genomic loci) to T2D [226], 41 genetic variants to fasting glucose [227], and 17 genetic variants to fasting insulin [227].

GWAS have made substantial progress in identifying genetic risk factors and understanding the mechanisms underlying T2D. Several genetic loci highly influence the risk of T2D. Large-scale GWAS have been highly effective in identifying of over 400 T2D-associated genetic variants, explaining the heritability of the disease by up to 20% [226,228,229], which offer mechanistic insights into T2D pathophysiology. Initially, most T2D-associated loci in populations of European ancestry were revealed back in 2007 [230,231]. Following efforts to identify additional T2D loci in the populations of Europe’s ancestry, large-scale meta-analyses of single studies [232,233,234] were carried out. For example, the DIAGRAM-DIAMANTE (Diabetes Genetics Replication and Meta-Analysis Consortium-Diabetes, Meta-Analysis Trans-Ethnic) consortium has meta-analysed 32 T2D GWAS of European descent participants (74,124 T2D cases and 824,006 controls) and discovered 243 T2D risk-associated genomic loci containing a total of 403 distinct (LD-independent) genetic risk variants [226]. The sample size was more than three times larger than in any previous T2D GWAS. Large-scale investigations increased our understanding of the genetic basis of T2D. With the emergence of the T2D genetic association dataset in a non-European ancestry group, trans-ancestry meta-analysis demonstrated a higher common susceptibility among worldwide populations. Most of the T2D loci are associated mainly with insulin production and the β-cell function with a significantly smaller number of genetic variants which seemingly affect insulin resistance. A large-scale meta-analysis of GWAS has been carried out using several cohorts with insulin sensitivity, processing, and secretion measurements to detect novel genetic variants related to insulin resistance. This technique has confirmed that several loci have been associated with certain glucose-related traits while also identifying novel loci. The Meta-Analyses of Glucose and Insulin-related Traits Consortium (MAGIC) conducted meta-analyses on GWAS from nondiabetic cohorts, including 151,188 individuals with fasting glucose (FG) measurements and 105,056 individuals with fasting insulin (FI) measurements, where they reported 41 genetic variants significantly associated with fasting glucose and 17 genetic variants significantly associated with fasting insulin [227].

Several GWAS studies on migraine have been conducted [223,224,225,235,236]. Between 2013 and 2016, the number of migraine risk loci increased from 13 [224] to 44 [223] as the number of samples and worldwide collaboration expanded. The 2016 meta-analysis of 22 GWAS studies included 59,674 cases and 316,078 controls, and identified 38 genome loci containing 44 independent genetic variants (SNPs) significantly associated with the risk of migraine [223]. Most of these common SNPs discovered were either intronic or intergenic, which is consistent with the assumption that most GWAS risk variants have regulatory impacts on gene expression rather than protein structure disruption. Analyses of the genes at the migraine risk loci indicated that the genes were enriched in vascular tissues and suggested a possible vascular mechanism of migraine [223]. Nitric oxide signalling and oxidative stress with the contribution of loci near *REST*, *GJA1*, *YAP1*, *PRDM16*, *LRP1*, and *MRVI1* also seem to be other significant pathways [223]. In the latest IHGC GWAS [225], 102,084 migraine cases and 771,257 controls were analysed to identify 170 index variants which map into 123 specific genomic risk loci, 86 of which have not been published previously. This latest GWAS performed multiple and more extensive tissue enrichment analyses and found genetic evidence for the function of both vascular and neuronal tissue types in migraine that refined earlier studies on smaller GWAS and tissue datasets. Identifying risk loci that encode migraine-specific targets were particularly fascinating findings in their GWAS. For example, a new GWAS locus on chromosome 11 contains the *CALCA* and *CALCB* genes which encode the alpha and beta calcitonin gene-related peptide (CGRP) isoforms, respectively [225].

CGRP is an extremely powerful vasodilator with a possible involvement in the pathophysiology of migraine headache [225]. Several biological migraine treatments against CGRP or its receptor were recently developed. CGRP-based monoclonal antibodies have notably been used to prevent migraines, and they have been seen as a significant achievement in migraine-specific therapy [225]. Interestingly, CGRP and related peptide amylin are located in the pancreas, where their role is to influence insulin secretion from the β-cells [237]. This process is significant as insulin regulates blood sugar levels by helping other body cells absorb or use glucose for energy. This procedure is disrupted in T2D when cells become insulin resistant, resulting in reduced absorption of insulin and higher blood sugar levels. The relationship between CGRP and glucose homeostasis is complicated. Research studies performed in rats with obesity and T2D revealed that the infusion of pharmaceutical doses of CGRP caused insulin resistance and lowered peripheral glucose clearance [101]. These findings all emphasise potential links between CGRP, the pathophysiology of migraines and glucose metabolism [238]. However, further research is needed to explain the effects of peptides on migraine and glucose-related traits.

In a recent GWAS studies of 102,084 migraineurs [225] and 74,124 T2D patients [226], the SNP rs1472662 [nearest coding gene *MACF1* (microtubule actin crosslinking factor 1)] and rs42854 (nearest coding gene *ANKDD1B* (ankyrin repeat and death domain containing 1B)] were found to be significantly associated (*p* < 5 × 10^−8^) with migraine and T2D and four SNPs were associated with migraine and T2D at genome-wide suggestive level. At the gene level, two genome-wide significant genes [*MACF1* and *THADA* (thyroid adenoma associated)] were associated with migraine and T2D. In addition, from the same migraine GWAS study, eight migraine risk loci (rs12598836, rs7618883, rs1472662, rs10894756, rs6693567, rs7916911, rs10866704, and rs843215) were also associated with fasting glucose [227], and six migraine loci (rs11165300, rs6668908, rs28455731, rs13235543, rs4739105, and rs4814864) were associated with fasting insulin [227] at a nominal *p*-value level (*p* < 0.05) (Table 2). The genetic risk loci for migraine and glucose-related traits implicated by GWAS are both new and recent and have not previously been examined via CGAS. However, our inquiry of the publicly available glucose-related traits GWAS summary data of European descent suggests that some of the migraine risk loci identified in the 2022 IHGC migraine GWAS [225] are also associated with FG [227], FI [227], and T2D [226] (Table 2). Table 2 summaries the genome-wide significant loci of migraine associated with glucose-related traits. 

## 4. Conclusions

Our review of previous studies highlighted several genes and biochemical pathways that could be shared between migraine and glucose-related traits. Candidate gene association studies of strong biological candidate genes have reported suggestive but inconsistent evidence for association with migraine and glucose-related traits. Impaired glucose metabolism, reduced insulin sensitivity, and fluctuation of blood glucose levels have all been associated with the comorbidity of migraine and glucose-related traits. Twin and family studies have demonstrated a strong genetic component for migraine and glucose-related traits. Two genome-wide significant loci associated with migraine (rs1472662 near *MACF1* and rs42854 near *ANKDD1B*) have also been associated with T2D at the genome-wide significant level, and two genes (*MACF1* and *THADA*) have been associated with both migraine and T2D at the gene-based genome-wide significant level. It is worth mentioning that GWAS has found *MACF1* to be associated with vascular diseases like hypertension and peripheral artery disease [239], further supporting the idea that headaches may somehow be linked to the vascular system. Additional migraine GWAS risk loci have been associated with fasting glucose and fasting insulin at (*p* < 0.05). These observations warrant further examination.

Among the investigated candidate gene polymorphisms, the *MTHFR* C677T, *TNF* −308(G/A), *NOS3* Glu298Asp, and *NOS3* [VNTR (27 bp) in intron 4] provided the most consistent evidence for association with both migraine and glucose-related traits, whereas genetic variants in the *INSR*, *ESR1*, and *PON1* candidate genes were less consistent. Both GWAS and candidate gene approaches have identified genes involved in the aetiology of migraine and glucose-related traits. For example, a recent GWAS study identified SNP rs17175860 within *INSR* significantly (*p* = 3 × 10^−14^) associated with T2D (www.ebi.ac.uk/gwas/, accessed on 13 March 2022) [229]. Additionally, SNP rs3845843 15,508 bp upstream of the TNF alpha-induced protein 6 gene (*TNFAIP6*) was significantly (*p* = 3 × 10^−11^) associated with an increased risk of T2D (www.ebi.ac.uk/gwas/, accessed on 13 March 2022) [229]. Notably, the TNF gene is involved in obesity-related insulin resistance [240] and was also associated with T1D at genome-wide suggestive level (rs3093664, *p* = 3.0 × 10^−7^) (www.ebi.ac.uk/gwas/, accessed on 13 March 2022) [241]. Studies have suggested that the link between migraine and diabetes may be caused by insulin resistance, which plays a vital role in developing metabolic syndrome and is also a risk factor for migraine [26,107]. A GWAS study conducted in 715 patients with Han Chinese ethnicity in Taiwan discovered that one SNP (rs146094041) in the Oestrogen related receptor gamma gene (*ESRRG*) was genome-wide significantly associated (*p* = 3.40 × 10^−9^) with migraine onset before the age of 12 years (www.ebi.ac.uk/gwas/, accessed on 13 March 2022) [242]. Given the involvement of sex hormones, especially oestrogen, in migraine pathophysiology [107,243], the link between migraine and diabetes may be partially explained by sex hormones. However, it is important to note that the remaining candidate genes reviewed here were not found to be associated with migraine or glucose-related traits in GWAS at the genome-wide significant level or genome-wide suggestive level. A similar finding was reached by de Vries et al. [244], who reported no experiment-wide significant results for the identified candidate genes previously associated with migraine. The most plausible explanations for GWAS findings not supporting previously implicated candidate genes relates to the large multiple test burden in GWAS, and the often small sample sizes utilised in CGAS—which increases the likelihood of producing both false-positive and false-negative results. In addition, over 90% of the genetic variants discovered by GWAS are in the non-coding regions and hence cannot be easily linked to a probable candidate gene [245].

This review provides insight into potential pleiotropic genes and shared biological mechanisms that may contribute to migraine and glucose-related traits; however, comprehensive cross-disorder investigations, utilising large and powerful genetic datasets, are required to understand how genetic risk factors mediate the relationship between migraine and glucose-related traits. Moreover, identifying shared genetic factors, and characterising their relationship in migraine risk and glucose-related traits, will enhance our understanding of their underlying biological mechanisms and enable the development of new biomarkers, new therapeutic targets, and tailor treatment strategies (e.g., glucose-related) in migraine patients.

## Figures and Tables

**Table 1:** A summary of candidate genes commonly associated with both migraine and glucose-related traits.

Candidate Genes	Function of Relative Protein	Polymorphisms	Migraine		Glucose-Related Traits	
			Supporting Association (Reference)	Not Supporting Association (Reference)	Supporting Association (Reference)	Not Supporting Association (Reference)
**Methylenetetrahydrofolate reductase (*MTHFR*)**	MTHFR enzyme transforms 5, 10-methylene tetrahydrofolate to 5-methyl tetrahydrofolate, an essential part of folate and homocysteine metabolism	C677T	[142,143,144]	[145,146]	[147,148,149]	[150,151]
**Insulin receptor (*INSR*)**	INSR mediates insulin’s activity on target cells and plays an important function in the regulation of glucose homeostasis	Val985Met, other polymorphisms	[72,73]	[152,153]	[154,155]	[156]
**Tumour necrosis factor (*TNF*)**	TNF initiates and regulates the chain of events that leads to an inflammatory response	−308 (G/A)	[142,157]		[158,159,160]	[161]
		−238 (A/G)			[162]	[163,164]
**Estrogen receptor 1 (*ESR1*)**	ESR1 has expressed variety of different tissues and organs, including vascular endothelial cells and trigeminal neurones with the linked with hormone system	Pvull (−397 T>C, rs2234693) and Xbal (−351 A>G, rs9340799)			[165]	[166,167]
		G594A	[168,169,170]	[171]		
**Nitric oxide synthase 3 (*NOS3*)**	NOS3 synthesise nitric oxide from L-arginine, a crucial endogenous endothelial-derived relaxing factor	VNTR (27 bp) in intron 4	[172,173]	[174]	[175,176,177]	
		Glu298Asp	[178,179]	[172]	[180,181]	
**Paraoxonase 1 (*PON1*)**	Enzyme involved in preventing LDL oxidation and endothelial dysfunction	Gln192Arg	[182]	[183,184]	[185,186,187,188]	

**Table 2:** Genome-wide significant migraine risk loci and their associations with FG, FI, and T2D.

						2022 IHGC Migraine		2018 DIAGRAM T2D		2021 MAGIC FG		2021 MAGIC FI	
Genes	SNP	CHR	BP	EA	NEA	OR	*p*-Value	OR	*p*-Value	OR	*p*-Value	OR	*p*-Value
*PRDM16*	rs10218452	1	3,075,597	G	A	1.12	7.26 × 10^−71^	0.99	0.27	1.00	0.450	1.00	0.985
*CAMTA1*	rs10128028	1	7,055,843	T	C	1.03	7.66 × 10^−9^	0.99	0.047	1.00	0.118	1.00	0.625
*TMEM51*	rs12057629	1	15,538,493	C	T	1.04	9.38 × 10^−14^	0.99	0.4	1.00	0.666	1.00	0.999
*INPP5B*	rs28739509	1	38,366,907	C	T	1.04	2.64 × 10^−10^	1.00	0.6	1.00	0.381	1.00	0.446
*C1orf87*	rs11578492	1	60,529,980	C	A	1.03	6.25 × 10^−9^	-	-	1.00	0.920	1.00	0.814
near *LEPR*	rs7511672	1	66,178,918	G	A	1.03	1.43 × 10^−9^	0.99	0.17	1.00	0.681	1.00	0.388
near *RP4-598G3.1*	rs56019088	1	73,891,226	I	D	1.05	7.32 × 10^−13^	-	-	1.00	0.765	1.00	0.708
*TGFBR3*	rs11165300	1	92,177,663	G	T	1.03	4.72 × 10^−8^	1.00	0.84	1.00	0.344	**0.99**	**0.035**
near *TSPAN2*	rs2078371	1	115,677,183	C	T	1.11	5.87 × 10^−42^	0.97	0.0025	1.01	0.130	0.99	0.230
*MEF2D*	rs2274319	1	156,450,873	T	C	1.08	2.74 × 10^−41^	1.01	0.42	1.00	0.562	0.99	0.052
*RABGAP1L*	rs11487328	1	174,601,659	G	C	1.05	1.70 × 10^−8^	-	-	1.00	0.633	1.00	0.689
*PLA2G4A*	rs6668908	1	186,913,055	G	T	1.03	2.22 × 10^−8^	1.01	0.22	1.00	0.629	**1.01**	**0.045**
near *MAPKAPK2*	rs56140113	1	206,843,108	C	T	1.04	7.76 × 10^−9^	1.00	0.95	0.99	0.195	1.00	0.638
*KIF26B*	rs72764846	1	245,847,455	G	A	1.04	5.41 × 10^−9^	1.01	0.14	-	-	-	-
*MACF1*	rs1472662	1	39,590,409	T	G	1.04	1.75 × 10^−8^	**1.08**	**1.60 × 10^−22^**	**1.01**	**0.011**	1.01	0.075
near *ADAMTSL4*	rs6693567	1	150,510,660	C	T	1.04	1.25 × 10^−13^	0.98	0.011	**1.01**	**0.032**	1.00	0.510
*THADA*	rs12712881	2	43,649,780	A	C	1.03	3.50 × 10^−10^	1.03	1.50 × 10^−7^	1.01	0.131	1.00	0.865
*ANKRD36C*	rs4907224	2	96,576,609	A	T	1.04	1.63 × 10^−9^	1.00	0.86	1.00	0.877	1.00	0.410
*ZEB2*	rs7564469	2	145,258,445	C	T	1.04	5.06 × 10^−9^	1.03	0.00064	1.00	0.360	1.00	0.247
near *AC064865.1*	rs895219	2	146,037,564	C	T	1.04	3.74 × 10^−11^	0.99	0.065	1.00	0.260	0.99	0.097
*MYO3B*	rs4668251	2	171,234,235	G	C	1.03	7.58 × 10^−9^	1.01	0.26	1.00	0.901	1.00	0.505
near *HOXD10*	rs72923449	2	176,978,383	C	A	1.08	4.66 × 10^−8^	1.02	0.23	-	-	-	-
*CARF*	rs138556413	2	203,832,867	C	T	1.14	4.15 × 10^−16^	1.03	0.12	-	-	-	-
near *TRPM8*	rs10166942	2	234,825,093	T	C	1.10	9.35 × 10^−51^	0.99	0.26	1.00	0.273	1.00	0.754
near *RNU6-546P*	rs843215	2	156,416,638	G	A	1.03	2.61 × 10^−8^	1.01	0.058	**1.01**	**0.042**	1.00	0.719
*ATRIP*	rs7618883	3	48,498,456	T	A	1.03	4.16 × 10^−8^	0.99	0.13	**0.99**	**0.010**	0.99	0.074
near *TGFBR2*	rs7371912	3	30,472,786	A	G	1.04	1.06 × 10^−14^	-	-	1.00	0.800	1.00	0.746
near *HNRNPA3P8*	rs950570	3	80,302,512	T	C	1.06	1.30 × 10^−8^	0.98	0.14	1.00	0.704	1.00	0.984
near *CADM2*	rs73138150	3	86,149,109	T	A	1.03	1.95 × 10^−8^	0.99	0.041	1.00	0.349	1.00	0.609
near *C3orf38*	rs6795209	3	88,210,464	A	G	1.04	1.23 × 10^−8^	-	-	1.00	0.458	0.99	0.123
*ITGB5*	rs1499963	3	124,607,055	C	T	1.03	7.48 × 10^−9^	0.99	0.059	1.00	0.293	1.00	0.770
near *GPR149*	rs13078967	3	154,289,946	A	C	1.16	2.16 × 10^−16^	1.00	0.95	-	-	-	-
near *SEC63P2*	rs73805934	4	35,469,918	G	C	1.04	1.11 × 10^−9^	1.00	0.93	1.00	0.708	1.00	0.975
near *SPINK2*	rs7684253	4	57,727,311	T	C	1.04	4.21 × 10^−14^	1.00	0.79	1.00	0.404	1.01	0.318
*ANKDD1B*	rs42854	5	74,963,277	G	C	1.04	9.40 × 10^−13^	**0.95**	**3.80 × 10^−13^**	1.01	0.221	0.99	0.057
near *SSBP2*	rs12653216	5	81,129,663	T	C	1.04	8.08 × 10^−9^	0.99	0.12	1.00	0.760	1.00	0.672
near *ZNF474*	rs11957829	5	121,515,195	G	A	1.04	1.58 × 10^−9^	0.99	0.41	1.00	0.839	1.00	0.885
*SNX24*	rs246326	5	122,306,398	T	C	1.05	6.80 × 10^−10^	1.01	0.15	1.00	0.807	1.00	0.617
near *POU4F3*	rs10038882	5	145,752,008	T	C	1.04	1.33 × 10^−12^	0.99	0.084	1.00	0.618	1.01	0.284
*TIGD6|HMGXB3*	rs4705403	5	149,380,493	A	G	1.05	1.18 × 10^−8^	1.00	0.68	1.00	0.354	1.00	0.877
near *NKX2-5*	rs6556059	5	172,645,766	T	C	1.03	8.16 × 10^−10^	1.00	0.48	0.99	0.158	1.01	0.286
*NSD1*	rs10866704	5	176,676,461	A	T	1.04	2.10 × 10^−8^	-	-	**1.01**	**0.039**	1.00	0.800
*PHACTR1*	rs9349379	6	12,903,957	A	G	1.08	1.41 × 10^−47^	1.00	0.83	1.00	0.415	1.00	0.132
near *PRL*	rs9295536	6	22,131,929	C	A	1.04	7.75 × 10^−12^	1.01	0.34	1.00	0.812	1.00	0.126
near *IER3*	rs9468830	6	30,749,712	T	G	1.04	2.38 × 10^−8^	1.00	0.97	1.00	0.909	1.00	0.227
*EHMT2*	rs74434374	6	31,850,308	C	A	1.08	4.52 × 10^−9^	0.99	0.55	-	-	-	-
*KCNK5*	rs10456100	6	39,183,470	T	C	1.05	9.16 × 10^−19^	0.97	8.90 × 10^−5^	1.00	0.970	1.00	0.427
near *KRT19P1*	rs34273564	6	72,321,017	T	C	1.03	1.00 × 10^−10^	1.00	0.48	0.99	0.178	1.00	0.658
*FHL5*	rs11153082	6	97,059,666	G	A	1.09	7.26 × 10^−54^	1.00	0.68	1.00	0.689	1.00	0.974
*REV3L*	rs6568677	6	111,713,302	A	G	1.04	2.09 × 10^−8^	1.02	0.0047	1.00	0.559	1.00	0.783
near *GJA1*	rs28455731	6	121,846,038	T	G	1.07	8.82 × 10^−23^	1.00	0.75	1.00	0.614	**0.98**	**0.027**
near *PCMT1*	rs9383843	6	150,133,954	C	A	1.03	1.35 × 10^−9^	1.01	0.047	1.00	0.355	1.00	0.983
*SUGCT*	rs10234636	7	40,427,617	T	C	1.09	4.43 × 10^−28^	0.99	0.33	1.00	0.743	1.00	0.413
*MLXIPL*	rs13235543	7	73,013,901	C	T	1.06	3.06 × 10^−13^	0.97	0.00038	1.00	0.419	**0.98**	**0.003**
*TSPAN12*	rs56067931	7	120,481,569	C	T	1.04	4.83 × 10^−8^	1.00	0.67	1.00	0.479	1.00	0.858
*PTK2B*	rs11782789	8	27,266,287	A	T	1.04	3.03 × 10^−9^	0.99	0.18	1.00	0.139	1.00	0.510
near *RP11-573J24.1*	rs4739105	8	64,496,159	T	C	1.04	2.85 × 10^−8^	1.01	0.11	1.00	0.542	**0.99**	**0.023**
*NFIB*	rs580845	9	14,103,618	A	C	1.03	4.30 × 10^−8^	0.99	0.31	1.00	0.778	1.00	0.226
near *RP11-373A6.1*	rs10156578	9	29,372,501	C	G	1.04	3.34 × 10^−12^	1.01	0.026	1.00	0.430	1.00	0.822
*TJP2*	rs7034179	9	71,746,838	T	C	1.04	1.60 × 10^−16^	1.00	0.77	0.99	0.496	1.01	0.684
*ZNF462*	rs17723637	9	109,687,403	G	A	1.04	8.63 × 10^−9^	1.00	0.63	1.00	0.427	1.00	0.226
*ASTN2*	rs3891689	9	119,258,583	C	T	1.06	2.28 × 10^−21^	1.00	0.88	1.01	0.087	1.00	0.887
near *EHMT1*	rs4278223	9	140,743,200	T	A	1.05	6.24 × 10^−10^	1.02	0.036	-	-	-	-
near *R-5SP299*	rs7916911	10	8,722,944	T	G	1.04	3.18 × 10^−12^	1.00	0.81	**0.99**	**0.035**	1.00	0.472
near *MLLT10*	rs10828247	10	21,822,856	G	A	1.03	7.51 × 10^−9^	1.00	0.87	1.00	0.834	1.00	0.561
*PLCE1*	rs2274224	10	96039597	G	C	1.06	3.28 × 10^−26^	1.00	0.76	1.00	0.648	1.00	0.113
*HPSE2*	rs12260159	10	100,702,737	G	A	1.09	7.33 × 10^−16^	-	-	1.00	0.459	1.00	0.846
*CNNM2*	rs12260436	10	104,741,114	C	A	1.04	7.29 × 10^−10^	0.99	0.12	1.00	0.786	1.00	0.581
*RBM20*	rs869432	10	112,502,662	A	C	1.03	3.54 × 10^−8^	1.02	0.0059	-	-	-	-
*HTRA1*	rs2672592	10	124,230,750	T	G	1.04	1.22 × 10^−12^	1.00	0.9	1.00	0.567	0.99	0.272
near *GPR26*	rs11248546	10	125,242,283	C	T	1.04	1.59 × 10^−12^	1.01	0.19	1.00	0.338	1.00	0.435
*INPP5A*	rs200314499	10	134,479,675	-	-	-	-	-	-	-	-	-	-
near *SPATA19*	rs10894756	11	133,745,852	G	A	1.03	2.83 × 10^−8^	1.00	0.51	**1.01**	**0.018**	1.00	0.890
*MRGPRE*	rs12295710	11	3,249,984	T	C	1.05	2.86 × 10^−16^	1.00	0.89	-	-	-	-
*MRVI1*	rs4910165	11	10,674,044	G	C	1.06	1.09 × 10^−24^	1.02	0.00094	1.00	0.363	1.00	0.370
near *INSC*	rs1003194	11	15,126,085	A	G	1.03	2.43 × 10^−10^	0.99	0.13	1.00	0.792	1.00	0.393
*MPPED2*	rs11031122	11	30,547,438	C	T	1.04	6.91 × 10^−10^	1.02	0.0031	1.00	0.122	1.00	0.669
*AMBRA1*	rs7932866	11	46,548,094	A	G	1.04	2.38 × 10^−9^	1.00	0.58	0.99	0.151	1.00	0.435
near *RAB3IL1*	rs12787928	11	61,697,078	A	T	1.03	6.85 × 10^−9^	0.99	0.11	1.00	0.615	0.99	0.084
*RBM14-RBM4|RBM4*	rs566673	11	66,401,373	G	T	1.03	9.07 × 10^−9^	1.01	0.1	1.00	0.955	1.00	0.713
*YAP1*	rs12226331	11	102,070,976	T	A	1.04	1.92 × 10^−13^	1.01	0.37	1.00	0.090	1.00	0.472
near *FGF6*	rs2160875	12	4,527,322	C	T	1.07	2.72 × 10^−36^	1.00	0.91	1.00	0.316	1.00	0.413
*PDZRN4*	rs1458170	12	41,901,277	C	T	1.04	5.75 × 10^−9^	0.98	0.016	1.00	0.187	1.00	0.929
*LRP1*	rs11172113	12	57,527,283	T	C	1.11	1.38 × 10^−90^	0.99	0.15	1.00	0.219	1.00	0.469
*ATP2B1*	rs4842676	12	90,091,782	C	G	1.04	2.26 × 10^−9^	0.99	0.34	1.00	0.391	1.00	0.380
near *RP11-690J15.1*	rs10777902	12	98,498,223	A	C	1.03	1.25 × 10^−10^	0.99	0.12	1.00	0.914	1.00	0.483
*NCOR2*	rs1271309	12	124,820,705	G	A	1.04	3.74 × 10^−8^	1.03	0.0035	0.99	0.246	1.00	0.851
*LRCH1*	rs7335684	13	47,193,696	G	A	1.03	1.05 × 10^−8^	0.99	0.035	1.00	0.845	1.00	0.810
*RNF219-AS1*	rs7996252	13	78,876,537	T	C	1.03	4.11 × 10^−8^	1.01	0.11	1.00	0.984	1.00	0.834
near *COL4A1*	rs2000660	13	110,788,441	A	G	1.05	4.95 × 10^−8^	1.00	0.77	-	-	-	-
near *RP11-384J4.2*	rs1245463	14	27,661,650	A	G	1.04	5.72 × 10^−14^	1.02	0.02	1.00	0.261	1.00	0.321
near *LRFN5*	rs1542668	14	42,548,912	G	A	1.03	2.53 × 10^−8^	1.01	0.46	1.00	0.450	1.01	0.095
near *ARID4A*	rs28756401	14	58,761,912	G	A	1.03	6.40 × 10^−9^	0.97	9.70 × 10^−7^	1.00	0.699	1.00	0.551
*DLST*	rs55707505	14	75,362,552	T	C	1.03	2.48 × 10^−8^	1.00	0.88	1.00	0.558	1.00	0.321
*IFT43*	rs75002882	14	76,496,477	G	T	1.17	9.22 × 10^−9^	0.97	0.25	-	-	-	-
near *ITPK1*	rs11624776	14	93,595,591	A	C	1.05	9.75 × 10^−19^	1.00	0.73	1.00	0.898	1.00	0.674
*SERPI-1*	rs28929474	14	94,844,947	T	C	1.12	2.54 × 10^−9^	0.90	5.90 × 10^−6^	-	-	-	-
*ABHD17C*	rs12708529	15	81,022,364	A	G	1.04	8.11 × 10^−10^	1.02	0.022	0.99	0.185	1.01	0.162
*HMOX2*	rs12598836	16	4,534,482	G	A	1.04	2.21 × 10^−10^	-	-	**1.01**	**0.008**	1.00	0.787
*CFDP1*	rs8046696	16	75,442,143	T	G	1.04	4.76 × 10^−14^	1.00	0.62	1.00	0.398	1.00	0.919
near *ZCCHC14*	rs8052831	16	87,578,039	G	A	1.04	8.25 × 10^−15^	1.01	0.07	1.00	0.792	1.01	0.123
*SMG6*	rs9894634	17	1,967,501	C	T	1.03	9.64 × 10^−11^	1.02	0.0041	1.00	0.921	1.00	0.454
*ZBTB4*	rs34914463	17	7,366,619	T	C	1.05	2.41 × 10^−9^	0.99	0.14	-	-	-	-
*HOXB3*	rs11652860	17	46,632,679	G	C	1.03	1.07 × 10^−8^	1.01	0.15	-	-	-	-
*RP11-81K2.1*	rs2119930	17	47,514,039	G	T	1.04	6.69 × 10^−15^	1.00	0.48	1.00	0.910	1.00	0.782
*MRC2*	rs12452590	17	60,720,058	G	T	1.04	2.03 × 10^−10^	0.97	2.50 × 10^−6^	-	-	-	-
*TBC1D16*	rs1285294	17	77,925,681	C	T	1.03	4.32 × 10^−8^	1.02	0.0064	1.01	0.075	1.00	0.289
*RNF213*	rs8077768	17	78,256,432	C	T	1.04	9.32 × 10^−13^	1.00	0.52	-	-	-	-
near *RBBP8*	rs7506921	18	20,201,527	A	T	1.04	1.17 × 10^−11^	-	-	1.00	0.546	1.00	0.748
near *SKOR2*	rs1019990	18	44,866,736	C	T	1.04	1.00 × 10^−11^	1.00	0.66	1.00	0.181	1.00	0.716
near *FECH*	rs8087942	18	55,192,245	A	G	1.04	9.71 × 10^−13^	1.00	0.8	1.00	0.906	1.00	0.946
*CAC-1A*	rs10405121	19	13,339,128	G	A	1.03	4.74 × 10^−10^	1.00	0.58	1.00	0.865	1.00	0.758
*SUGP1*	rs74182632	19	19,406,126	A	G	1.07	1.43 × 10^−8^	1.01	0.36	1.01	0.410	0.98	0.054
*B9D2/TMEM91*	rs1982072	19	41,864,509	A	T	1.04	4.22 × 10^−11^	1.01	0.22	1.00	0.773	1.00	0.279
near *JAG1*	rs111404218	20	10,684,159	-	-	-	-	-	-	-	-	-	-
*SLC24A3*	rs4814864	20	19,469,817	C	G	1.07	1.44 × 10^−28^	0.99	0.47	1.00	0.638	**1.01**	**0.031**
*C20orf112*	rs6057599	20	31,168,439	T	C	1.04	8.73 × 10^−14^	1.01	0.4	1.01	0.178	1.01	0.149
*ZMYND8*	rs910187	20	45,841,052	G	A	1.04	1.14 × 10^−10^	0.98	0.001	1.00	0.211	1.00	0.574
near *MRPS6*	rs28451064	21	35,593,827	G	A	1.07	3.52 × 10^−15^	1.02	0.031	-	-	-	-
*RUNX1*	rs764508	21	36,935,896	C	T	1.03	3.28 × 10^−9^	0.99	0.16	1.00	0.632	1.00	0.139
near *AC006547.14*	rs625686	22	20,142,932	C	T	1.03	8.26 × 10^−9^	0.99	0.042	1.00	0.430	1.00	0.720
near *FAM47A*	rs1507220	X	34,102,712	A	C	1.03	2.67 × 10^−8^	-	-	0.99	0.665	1.02	0.262
near *MED14*	rs4403550	X	40,746,484	-	-	-	-	-	-	-	-	-	-

Odds ratio (OR) and *p*-value associated with effect allele (EA); Non-effect allele (NEA); Chromosome (CHR), Base pair position (BP); Single nucleotide polymorphism (SNP).

## Data Availability

No new data were generated or analysed in this study. Data sharing was not applicable.
